# Identifying transcription factors that reduce wood recalcitrance and improve enzymatic degradation of xylem cell wall in *Populus*

**DOI:** 10.1038/s41598-020-78781-6

**Published:** 2020-12-16

**Authors:** Chiaki Hori, Naoki Takata, Pui Ying Lam, Yuki Tobimatsu, Soichiro Nagano, Jenny C. Mortimer, Dan Cullen

**Affiliations:** 1 Research Faculty of Engineering, Hokkaido University, Sapporo, 060-8628 Japan; 2 Forest Bio-Research Center, Forestry and Forest Products Research Institute, Forest Research and Management Organization, Hitachi, Ibaraki 319-1301 Japan; 3 Research Institute for Sustainable Humanosphere, Kyoto University, Uji, Kyoto, 611‐0011 Japan; 4 Forest Tree Breeding Center, Forestry and Forest Products Research Institute, Forest Research and Management Organization, Hitachi, Ibaraki 319-1301 Japan; 5 Environmental Genomics and Systems Biology Division, Lawrence Berkeley National Laboratory, Joint BioEnergy Institute, Berkeley, CA 94720 USA; 6 U. S. Department of Agriculture, Forest Products Laboratory, Madison, WI 53726 USA

**Keywords:** Plant biotechnology, Cell wall

## Abstract

Developing an efficient deconstruction step of woody biomass for biorefinery has been drawing considerable attention since its xylem cell walls display highly recalcitrance nature. Here, we explored transcriptional factors (TFs) that reduce wood recalcitrance and improve saccharification efficiency in *Populus* species. First, 33 TF genes up-regulated during poplar wood formation were selected as potential regulators of xylem cell wall structure. The transgenic hybrid aspens (*Populus tremula* × *Populus tremuloides*) overexpressing each selected TF gene were screened for in vitro enzymatic saccharification. Of these, four transgenic seedlings overexpressing previously uncharacterized TF genes increased total glucan hydrolysis on average compared to control. The best performing lines overexpressing *Pt* × *tERF123* and *Pt* × *tZHD14* were further grown to form mature xylem in the greenhouse. Notably, the xylem cell walls exhibited significantly increased total xylan hydrolysis as well as initial hydrolysis rates of glucan. The increased saccharification of *Pt* × *tERF123*-overexpressing lines could reflect the improved balance of cell wall components, i.e., high cellulose and low xylan and lignin content, which could be caused by upregulation of cellulose synthase genes upon the expression of *Pt* × *tERF123*. Overall, we successfully identified Pt × tERF123 and Pt × tZHD14 as effective targets for reducing cell wall recalcitrance and improving the enzymatic degradation of woody plant biomass.

## Introduction

Woody plants are the most abundant source of terrestrial biomass and have been industrially used for construction, paper, energy and many materials and chemicals. Recently, wood has also been considered as a promising sustainable resource for the production of biofuels and other high-value products^1^. However, the efficient conversion of lignocellulose into simple sugars (glucose and xylose) has been stymied by its complex and recalcitrant structures. Therefore, recent studies have focused on optimizing wood cell wall characteristics and traits for conversion processes including more efficient enzymatic deconstruction using gene modification and metabolic engineering^2^.

*Populus* species (poplars, aspens and cottonwoods) are excellent models of woody plant biomass because of their worldwide distribution, fast growth, genomic resources, and technical advances such as the well-established transformation system^3^. *Populus* stems include many different cell types which differentiate from the cambium into vessel elements, fibers, and axial and radial parenchyma cells. The dominant material in woody biomass is the secondary xylem cell wall of fiber cells. In these cells, a thin and stretchy primary cell wall (PCW) layer is first deposited during cell expansion. Afterwards, a thick and rigid secondary cell wall (SCW) layer is deposited inside the PCW layer. The PCW is mainly constituted of cellulose, xyloglucan, and pectin with negligible amount of lignin, whereas the SCW consists of cellulose, xylan, mannan and lignin^4,5^. Genes encoding enzymes involved in the biosynthesis of cell wall components such as cellulose, hemicelluloses and lignin have been relatively well characterized in *Populus* species (reviewed by Ye and Zhong^6^).

Expression of the cell wall biosynthetic enzymes is controlled by a number of transcriptional factors (TFs). NO APICAL MERISTEM/ARABIDOPSIS TRANSCRIPTION ACTIVATION FACTOR/CUP-SHAPED COTYLEDON (NAC) and MYB families genes play critical roles in regulating xylem cell differentiation and cell wall thickening^7,8^. Homologous genes of *Arabidopsis thaliana* VASCULAR-RELATED NAC DOMAIN (VND)^9^, NAC SECONDARY WALL THICKENING PROMOTING FACTOR/SECONDARY WALL-ASSOCIATED NAC DOMAIN (NST/SND)^10,11^ and SOMBRERO (SMB) proteins designated as VNS (VND, NST/SND,and SMB RELATED) proteins^12^ are key regulators of secondary cell wall formation in xylem fibers, phloem fibers, and xylem ray parenchyma cells in *Populus*^12–16^. Among these, VNS09, VNS10, VNS11 and VNS12 corporately play important roles as a master switch regulators of cell wall formation in *Populus*^17,18^, but their downstream genes including several TFs are functionally yet uncharacterized. In addition to these studies of *A. thaliana* TF homologs in *Populus* species, more comprehensive analyses have focused on TFs expressed during the wood formation processes including secondary xylem differentiation, cell expansion along with PCW deposition, SCW deposition, and programmed cell death along with further lignification^19–21^. Transcriptomic analyses revealed more than 1000 TF genes that were potentially involved in the development of secondary xylem cells^19,20^. However, most of TFs are uncharacterized, with the exception of major TF families such as NAC and MYB^22,23^. Furthermore, yet only a few studies have succeeded in improving biomass quality by manipulating *Populus* TF genes^24,25^. Thus, our understanding of the TFs involved in the regulatory network system of *Populus* xylem cell wall formation and the potential for application in a bioengineering context is still limited.

Toward reducing wood recalcitrance and improving its enzymatic degradability, we focused on TF genes associated with wood formation as xylem cell wall modification targets, since most previous studies have focused on genes involved in cell wall biosynthesis or cell wall-degrading/-modifying enzymes in *Populus*^26,27^. In comparison, TFs can affect the synthesis of multiple cell wall components and thereby have greater effects. We selected 33 candidate TF genes that are highly up-regulated during wood formation, over-expressed them in hybrid aspen, *Populus tremula* × *Populus tremuloides* (*Pt* × *t*, wild-type clone T89), and analyzed their saccharification properties.

## Results

### Selection of target TFs to manipulate xylem cell wall in hybrid aspen

To engineer xylem cell walls of hybrid aspen with improved enzymatic saccharification properties, first we explored the candidate TFs involved in xylem cell wall formation in *Populus* species. Targets were selected from genes which are downstream of a master switch regulator of SCW formation in *Populus*, VNS10^17^. Other targets were selected from genes spatially and temporally expressed during xylem formation^19^. As a result, a total of 33 TFs (Table 1) including 13 MYBs, 7 NACs and 2 TCPs were selected as target TFs for this study. A series of first and second master regulators such as NST/SNDs, VNDs, MYB002, and MYB021^22,23^ were eliminated from the target TFs, since these transcriptional activators were known to strongly modify SCW structures, affect plant growth negatively, and possibly lower biomass saccharification performance when overexpressed *in planta*^17^. Among the 33 TFs, MYB003 and MYB152 were characterized as transcriptional activators of SCW biosynthesis genes^28–30^, and MYB199 and KNAT7 were identified as transcriptional repressor for SCW formation in *Populus*^31,32^. The function of the other 29 TFs remains to be elucidated.

**Table 1:** List of transcription factors selected in this study.

Gene	Description	Gene name used in this study	Locus ID*** or Accession number	Locus ID in the genome database of *Populus trichocarpa*
*MYB003*	MYB domain protein 003	*Pt* × tMYB003*	Potrx065703g25822	Potri.001G267300
*MYB010*	MYB domain protein 010	*Pt* × tMYB010*	Potrx010292g08846	Potri.001G099800
*MYB026*	MYB domain protein 026	*Pt* × tMYB026*	Potrx009736g08025	Potri.005G063200
*MYB055*	MYB domain protein 055	PniMYB055**	AB970784	Potri.014G111200
*MYB090*	MYB domain protein 090	*Pt* × tMYB090*	Potrx065174g25289	Potri.015G033600
*MYB125*	MYB domain protein 125	*Pt* × tMYB125*	Potrx047741g14217	Potri.003G114100
*MYB148*	MYB domain protein 148	*Pt* × tMYB148*	Potrx006180g04741	Potri.012G084100
*MYB152*	MYB domain protein 152	PniMYB152**	AB970789	Potri.017G130300
*MYB158*	MYB domain protein 158	*Pt* × tMYB158*	Potrx065343g25445	Potri.005G186400
*MYB161*	MYB domain protein 161	*Pt* × tMYB161*	Potrx058176g19613	Potri.007G134500
*MYB192*	MYB domain protein 192	*Pt* × tMYB192*	Potrx007540g05840	Potri.007G067600
*MYB199*	MYB domain protein 199	*Pt* × tMYB199*	Potrx041107g12245	Potri.012G127700
*MYB212*	MYB domain protein 212	*Pt* × tMYB212*	Potrx063727g24046	Potri.008G101400
*TCL1*	TRICHOMELESS1, MYB-related protein	*Pt* × tTCL1*	Potrx065837g25958	Potri.002G168900
*PNAC052*	Populus NAC domain protein 52	*Pt* × tPNAC052*	Potrx008983g07023	Potri.014G041300
*PNAC055*	Populus NAC domain protein 55	*Pt* × tPNAC055*	Potrx065803g25919	Potri.014G025700
*PNAC069*	Populus NAC domain protein 69	*Pt* × tPNAC069*	Potrx007925g06145	Potri.009G141600
*PNAC122*	Populus NAC domain protein 122	PniPNAC122**	AB970793	Potri.007G135300
*PNAC124*	Populus NAC domain protein 124	*Pt* × tPNAC124*	Potrx052714g16378	Potri.011G058400
*PNAC128*	Populus NAC domain protein 128	*Pt* × tPNAC128*	Potrx007682g05931	Potri.018G068700
*PNAC161*	Populus NAC domain protein 161	*Pt* × tPNAC161*	Potrx056901g18742	Potri.003G022800
*TCP3*	TCP domain (bHLH) protein 3	*Pt* × tTCP3*	Potrx000869g00654	Potri.001G327100
*TCP24*	TCP domain (bHLH) protein 24	*Pt* × tTCP24*	Potrx042116g12507	Potri.013G119400
*KNAT7*	Homeobox knotted protein 7	PniKNAT7**	AB970798	Potri.001G112200
*HAT22*	Homeobox leucine zipper protein 22	*Pt* × tHAT22*	Potrx047905g14286	Potri.002G113400
*GATA8*	GATA (type-IV Zinc finger) protein 8	PniGATA8**	AB970779	Potri.008G038900
*DOF4.6*	DNA binding with one finger (C2H2) protein 4.6	*Pt* × tDOF4.6*	Potrx054398g17215	Potri.003G144500
*ZF1*	C2H2-type Zinc finger 1	PniZF1**	AB970797	Potri.017G091800
*ERF123*	Ethylene response factor (ERF) family protein 123	*Pt* × tERF123*	Potrx058048g19531	Potri.006G080300
*bZIP10*	Basic leucine-zipper protein 10	*Pt* × tbZIP10*	Potrx062638g23085	Potri.002G045800
*WLIM2B*	Widely expressed LIM domain protein	*Pt* × tWLIM2B*	Potrx063364g23686	Potri.010G193800
*LBD15*	Lateral organ boundaries (LOB) domain protein 15	*Pt* × tLBD15*	Potrx063009g23406	Potri.013G156200
*ZHD14*	Zinc finger homodomain protein 14	*Pt* × tZHD14*	Potrx005907g04556	Potri.004G126500

*Pt × t; *Populus tremula* × *Populus tremuloides.*

**Pni; *Populus nigra.*

*** http://popgenie.org.

To further characterize the 33 target TFs, their gene expression patterns during plant developments^33^ were analyzed. In short, almost all the TFs were highly expressed during xylem developmental processes in *Populus* (Figure S1). Next, using the AspWood database^21^, hierarchical cluster analysis of their gene expression during wood formation was analyzed together with 40 other cell wall synthesis-related genes^26,27^. This analysis revealed that the target TF genes were divided into three clusters (I, II and III; Figure S2). Cluster I contained 22 target TFs including previously characterized TFs such as Pt × tMYB003, Pt × tMYB152, Pt × tMYB199 and Pt × tKNAT7^28–32^. Members of cluster I were highly expressed during SCW formation and wood fiber maturation. Cluster I also included 25 SCW biosynthetic enzymes associated with cellulose biosynthesis [five CELLULOSE SYNTHASEs (CESAs); CESA4, CESA7-A, CESA7-B, CESA8-A and CESA8-B], hemicellulose biosynthesis [six GLYCOSYLTRANSFERASEs (GTs); GT8D-1/IRREGULAR XYLEM 8 (IRX8), GT43A/IRX9, GT43B/IRX9, GT43C/IRX14, GT47A-1/IRX10 and GT47C/ /FRAGILE FIBER 8 (FRA8)], or lignin biosynthesis [*p*-COUMARATE 3-HYDROXYLASE (C3′H1), CINNAMATE-4-HYDROXYLASEs (C4H1 and C4H2), CINNAMYL ALCOHOL DEHYDROGENASE (CAD1), CAFFEOYL-CoA *O*-METHYLTRANSFERASEs (CCoAOMT1, CCoAOMT2 and CCoAOMT3), CINNAMOYL-CoA REDUCTASE (CCR7), CAFFEIC ACID *O*-METHYLTRANSFERASEs (COMT1 and COMT2), FERULATE 5-HYDROXYLASEs (F5H1 and F5H2), and *p*-HYDROXYCINNAMOYL-CoA:QUINATE/SHIKIMATE *p*-HYDROXYCINNAMOYLTRANSFERASEs (HCT1 and HCT6)]. In cluster II, six TFs (Pt × tERF123, PniMYB055, Pt × tTCP24, Pt × tWLIM2B, Pt × tPNAC161, and Pt × tMYB148) were induced at the initial stage of the xylem development. These TFs showed similar gene expression patterns to 14 PCW-related genes associated with cellulose biosynthesis (CESA1-A, CESA1-B, CESA3-A, CESA6-A and CESA6-B), xyloglucan biosynthesis [XYLOSYLTRANSFERASE (XXT1A) and CELLULOSE SYNTHASE-LIKE C-R (CSLC-R)], or pectin biosynthesis [GALACTURONOSYLTRANSFERASEs (GAUT1A and GAUT1B), GALACTURONOSYLTRANSFERASE-LIKEs (GATL3A and GATL3B], GALACTOSYLTRANSFERASEs (GALS1,GALS2A, and GALS2B]. Five TFs (Pt × tDOF4.6, Pt × tMYB192, Pt × tPNAC069, Pt × tMYB212, and Pt × tTCP3) were categorized into cluster III, which were highly expressed at later stage of xylem development and phloem tissues.

### Screen of 33 TF-overexpressed poplar seedlings by enzymatic saccharification

**Figure 1:**
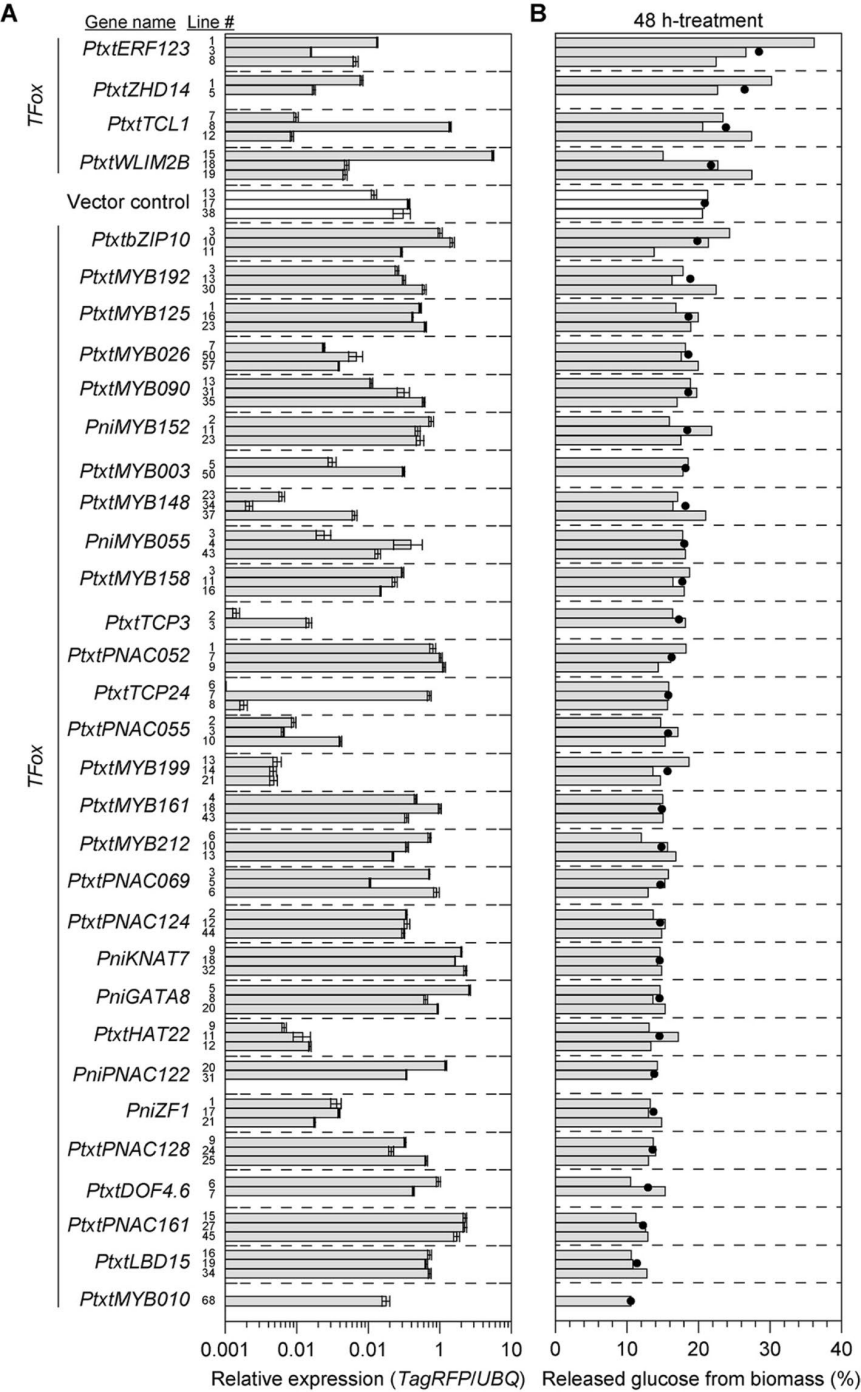
Relative expression level of the transgene (TF-TagRFP) in transgenic hybrid aspens measured by real-time PCR using primer pairs specific to the RFP (**A**). pGWB560 harboring the NLS-TagRFP was used as a vector control. TagRFP regions fused with each introduced gene as well as vector control were amplified. No amplicon was detected in wild type of T89 plants. Released glucose amounts from biomass (wt/wt %) of seedling stems of the transgenic hybrid aspens grown in MS culture under aseptic condition after 48 h-treatment with enzyme cocktail (**B**). A series of TFs were sorted by average of released glucose (%) from different lines generated from the same genotype (filled circle).

The selected 33 TFs, fused with TagRFP at the C-terminal, were overexpressed in hybrid aspens by using a pGWB560 vector harboring the nuclear localization sequence (NLS), and pGWB560 harboring NLS tagged double-TagRFP (NLS-TagRFP) was used as a vector control. Expression of the introduced genes was confirmed by detection of the TagRFP-tag with real-time PCR analysis. TagRFP region of vector control plants was also amplified; we confirmed that no amplicon was detected in the wild type of T89 plants. Relative expression (to *Pt* × *tUBQ*) of the introduced genes ranged from 0.001 to 5 as shown in Fig. 1A. The harvested seedling stems of the successful transgenic hybrid aspens grown in a MS culture under aseptic condition were treated with a cell-wall degrading enzyme cocktail for 48 h and the released glucose amounts were measured by colorimetric methods via glucose oxidase reactions to calculate saccharification efficiency (Fig. 1B).

Most of the tested transgenic aspens overexpressing a variety of TFs involved in SCW formation, such as MYB, NAC and KNOX proteins, showed a decline in the amount of released glucose compared to control. In contrast, four of the TF overexpressing lines exhibited increased release of glucose (Fig. 1B). All four lacked functional characterization in *Populus* previously: (1) Pt × tERF123ox overexpressing *ETHYLENE RESPONSE FACTOR 123* (*Pt* × *tERF123*)^34^ exhibited almost 1.5-times glucose release (28.45% ± 7.05, mean ± s.d.), compared to the control (20.90% ± 0.37); (2) Pt × tZHD14ox overexpressing *ZINC FINGER HOMEODOMAIN 14* (*Pt* × *tZHD14*)^35^ had ~ 1.3 times increase (26.45% ± 5.35) compared to the control; (3) Pt × tTCL1ox overexpressing *TRICHOMELESS 1* (*Pt* × *tTCL1*)^36^, a MYB-related protein (a single repeat R3 MYB protein apart from R2R3 MYB family described above) showed the average of glucose amounts released from biomass (23.84% ± 3.40), which were also greater than that of control. The different lines (#7, #8, and #12) showed 23.46%, 20.65% and 27.42%, respectively. Considering the relative expression levels of the corresponding lines (0.0098, 1.4033 and 0.0086, respectively), the high ectopic expression of *Pt* × *tTCL1* is likely the cause of the poor performance of line #8; (4) Pt × tWLIM2Box overexpressing *WIDELY EXPRESSED LIN-11, Isl1 and MEC-3 (LIM)-STRUCTURAL DOMAIN PROTEIN 2B* (*Pt* × *tWLIM2B*)^37^ showed a significant increase in two of the lines (#18 = 22.73% and #19 = 27.47%, respectively) but a significant decrease in line #15 (15.08%), which was also likely due to the high ectopic expression of *Pt* × *tWLIM2B*.

### Enzymatic saccharification of Pt × tERF123ox and Pt × tZHD14ox grown in a greenhouse

**Figure 2:**
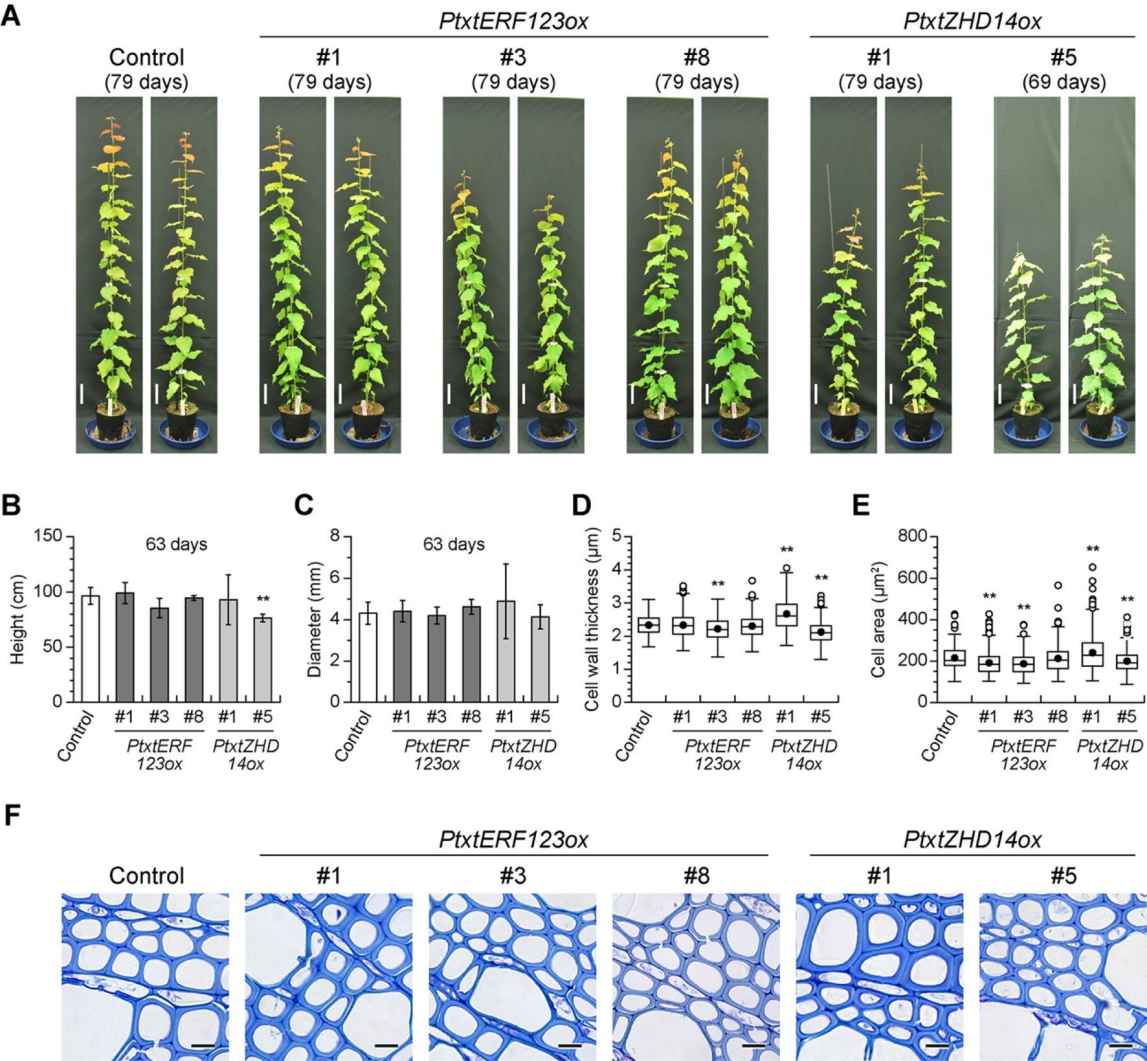
Growth of Pt × tERF123ox, and Pt × tZHD14ox lines grown in the soil. Phenotypes of 79- and 69-days-old transgenic hybrid aspens compared with the control (expressing GFP-TUA6 alone) (**A**). Scale bar = 10 cm. Height (**B**) and diameter (**C**) of transgenic hybrid aspens and control plants. Cell wall thickness (**D**) and cell size (**E**) of wood fiber cells. Three hundred fiber cells were estimated from three trees per each genotype. Single and double asterisk(s) indicate *P* value < 0.05 and < 0.01, respectively in Student’s *t*- test when compared with the control plants. Anatomical observations of the stem sections stained with toluidine blue (**F**). Scale bar = 10 μm.

To assess the enzymatic digestibility of mature xylem tissues, Pt × tERF123ox and Pt × tZHD14ox, the two transgenic aspen lines which showed the largest glucose releases in the initial saccharification screening as described above, were grown in a soil in a greenhouse with at least 3 biological replicates for further characterizations. In short, no significant phenotypic difference in stem heights and diameters was observed between the TF-overexpressing lines and the controls, except for Pt × tZHD14ox line #5 displaying apparently shorter stem heights than the controls (Fig. 2A–C). Based on anatomical stem sections stained with toluidine blue, xylem cell size and cell wall thickness were not changed between Pt × tERF123ox lines and the controls, except for Pt × tERF123ox line #3 (Fig. 2D–F). In case of Pt × tZHD14ox, compared to the controls, the xylem cell walls were thicker and thinner in line #1 and line #5, respectively (Fig. 2D–F). Overall, no apparent loss of biomass quantity was observed between the TF-overexpressing lines and the controls except for Pt × tZHD14ox line #5.

**Figure 3:**
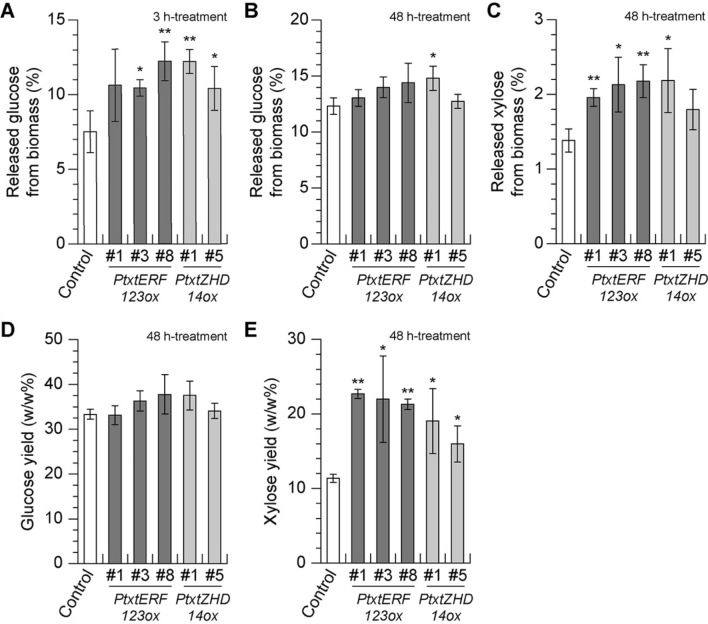
The enzymatic saccharification of Pt × tERF123ox, and Pt × t ZHD14ox lines grown in the soil, in comparison to the controls (GFP-TUA6). Released glucose (**A**,**B**) and xylose (**C**) amounts from biomass (wt/wt %) after 3 h (**A**) or 48 h (**B**,**C**)-treatments with cellulase cocktail. Glucose and xylose yields (**D**,**E**) calculated based on a total of glucan (non-crystalline glucan and crystalline glucan) and xylan measured in Table 2. Data are shown as mean ± standard deviations of biological triplicates. Single and double asterisk(s) indicate *P* value < 0.05 and < 0.01, respectively in Student’s *t*- test when compared with the control plants.

Consistent with the results obtained from the screening of seedling tissue (Fig. 1), the amount of glucose released from xylem biomass of both Pt × tERF123ox and Pt × tZHD14ox plants (except for Pt × tZHD14ox line #5) were increased after 48 h-treatment with the enzyme cocktail as compared to the controls (Fig. 3B). For Pt × tERF123ox, the descending order of the glucose release was line #8 > #3 > #1. In particular, the initial hydrolysis rates of the transgenic poplar biomass were significantly increased (Fig. 3A). In addition to glucose release, the amounts of xylose released after 48 h-treatment with the enzyme cocktail were also measured. Notably, xylose released from both Pt × tERF123ox and Pt × tZHD14ox biomass, again except for Pt × tZHD14ox line #5, were significantly increased (Fig. 3C). To further assess the enzymatic digestibility, released amount of glucose and xylose from a total of glucan (non-crystalline and crystalline glucan) and xylan (Table 2) were calculated as glucose yield and xylose yield (Fig. 3D,E), respectively. Interestingly, all the xylose yields of Pt × tERF123ox and Pt × tZHD14ox lines were approximately 1.5–2 times higher than the controls whereas the enhancement of glucose yield exhibited less significance. Collectively, these data suggested that overexpression of *Pt* × *tERF123* and *Pt* × *tZHD14* led to improved biomass digestibility most likely via alterations of xylem cell wall structure. Notably, there were some differences in the enzymatic digestion of the poplar seedling and mature xylem biomass tested. For example, glucose releases after 48 h enzymatic hydrolysis of the mature xylems were not increased as much as those of the seedlings. These different enzymatic saccharification performances between the seedling and mature xylem biomass might be due to the differences in the sample part analyzed (i.e., seedlings include xylem as well as phloem and pith whereas bark and pith were removed from mature xylem), xylem developmental stages and plant cultivation conditions (i.e., growth chamber vs greenhouse conditions).

**Table 2:** Cell wall lignin and polysaccharide analyses of Pt × tERF123ox and Pt × tZHD14ox transgenic hybrid aspens grown in green house.

Sample	Lignin and sugar content (mg/g CWR)								Lignin aromatic composition^d^			
	Lignin^a^	Arabinan	Xylan	Mannan	Galactan	Amorphous glucan^b^	Crystalline glucan^c^		S (%)	G (%)	H (%)	S/G ratio
Control	185.4 ± 6.0	3.3 ± 0.4	116.5 ± 12.8	7.0 ± 0.4	6.4 ± 0.3	9.5 ± 0.7	371.7 ± 0.6		60.6 ± 2.0	39.3 ± 2.0	Trace	1.55 ± 0.13
Pt × tERF123ox line #1	168.6 ± 2.2**	3.3 ± 0.2	86.4 ± 4.0*	6.2 ± 0.6	6.5 ± 0.4	9.7 ± 0.5	383.8 ± 7.6		61.3 ± 0.5	38.7 ± 0.5	Trace	1.65 ± 0.15
Pt × tERF123ox line #3	174.1 ± 7.6	4.1 ± 0.7	98.5 ± 12.2	5.9 ± 0.3*	7.1 ± 0.5	10.3 ± 1.0	375.5 ± 7.7		60.0 ± 1.1	39.9 ± 1.1	Trace	1.40 ± 0.05
Pt × tERF123ox line #8	173.6 ± 1.2*	3.7 ± 0.1	96.9 ± 5.0	6.9 ± 0.5	6.0 ± 1.7	9.8 ± 0.4	383.5 ± 14.2		57.9 ± 0.8	42.0 ± 0.8	Trace	1.58 ± 0.03
Pt × tZHD14 line #1	179.9 ± 2.7	3.3 ± 0.1	114.7 ± 7.2	5.6 ± 0.6*	7.3 ± 0.6	11.6 ± 0.3**	382.5 ± 5.7		61.8 ± 1.8	38.2 ± 1.8	Trace	1.51 ± 0.07
Pt × tZHD14 line #5	207.3 ± 5.6**	4.0 ± 0.1*	116.4 ± 8.4	5.9 ± 0.5*	7.4 ± 0.1**	10.8 ± 1.4	357.1 ± 2.7**		58.0 ± 0.8	41.5 ± 0.8	Trace	1.38 ± 0.05

Single and double asterisk(s) indicate *P* value < 0.05 and < 0.01, respectively, in Student’s *t*- test (*n* ≥ 3) when compared with the control plant.

*CWR* cell wall residues, *S* syringyl, *G* guaiacyl, *H* *p*-hydroxyphenyl.

^a^Determined by thioglycolic acid assay.

^b^Trifluoroacetic-acid-soluble glucan.

^c^Trifluoroacetic-acid-insoluble glucan.

^d^Determined by analytical thioacidolysis.

### Chemical composition of Pt × tERF123ox and Pt × tZHD14ox mature xylem cell walls

To investigate the cause of the enhanced saccharification performance of Pt × tERF123ox and Pt × tZHD14ox, lignocellulose composition and structures of their xylem cell wall samples were investigated by a series of chemical analyses (Table 2). Lignin content of the aspen cell wall samples were determined by thioglycolic acid assay^38^, and lignin composition, i.e., syringyl (S), guiacyl (G) and *p*-hydroxyphenyl (H) aromatic unit ratio, was determined by analytical thioacidolysis^39^. In addition, cell wall polysaccharide composition was determined by quantification of monomeric sugars released via a two-step acid-catalyzed hydrolysis method^39,40^.

The cell wall samples from two of the three Pt × tERF123ox lines, i.e., lines #1 and #8, showed significantly reduced lignin contents, by 9.1% and 6.4%, respectively, compared to the controls, whereas the other line #3 also showed a decreased lignin level, by 6.1%, albeit with no statistical significance. In addition, all the three Pt × tERF123ox lines showed tendencies toward decreased xylan and mannan, and increased cellulosic crystalline and amorphous glucan levels. No significant change in the S/G/H ratio was observed between all the Pt × tERF123ox and control cell wall samples (Table 2). Overall, these chemical analysis data suggested that the overexpression of Pt × tERF123 may lead to increased glucan and reduced xylan barrier and lignin recalcitrance and thereby boost enzymatic hydrolysis of both cellulose and xylan polysaccharides.

In contrast to the Pt × tERF123ox lines, the lignin content of the cell wall samples from the Pt × tZHD14ox lines appeared to be comparable or even higher than that of the control cell walls. Cellulosic crystalline glucan contents of the Pt × tZHD14ox cell walls seemed to be comparable or lower than that of the controls (Table 2). The two independent Pt × tZHD14ox lines, i.e., lines #1 and #5, both showed reduced mannan, and increased galactan and amorphous glucan levels. Meanwhile, similar to the Pt × tERF123ox lines, we observed no significant change in the S/G/H lignin unit ratio of the Pt × tZHD14ox cell walls compared to the control (Table 2). Collectively, in contrast to our observation with Pt × tERF123ox, the improved saccharification performance of Pt × tZHD14ox is not apparently associated with changes to the glucan, xylan and lignin contents of the cell wall.

### Expression of lignin and cellulose biosynthetic genes in Pt × tERF123ox and Pt × tZHD14ox

**Figure 4:**
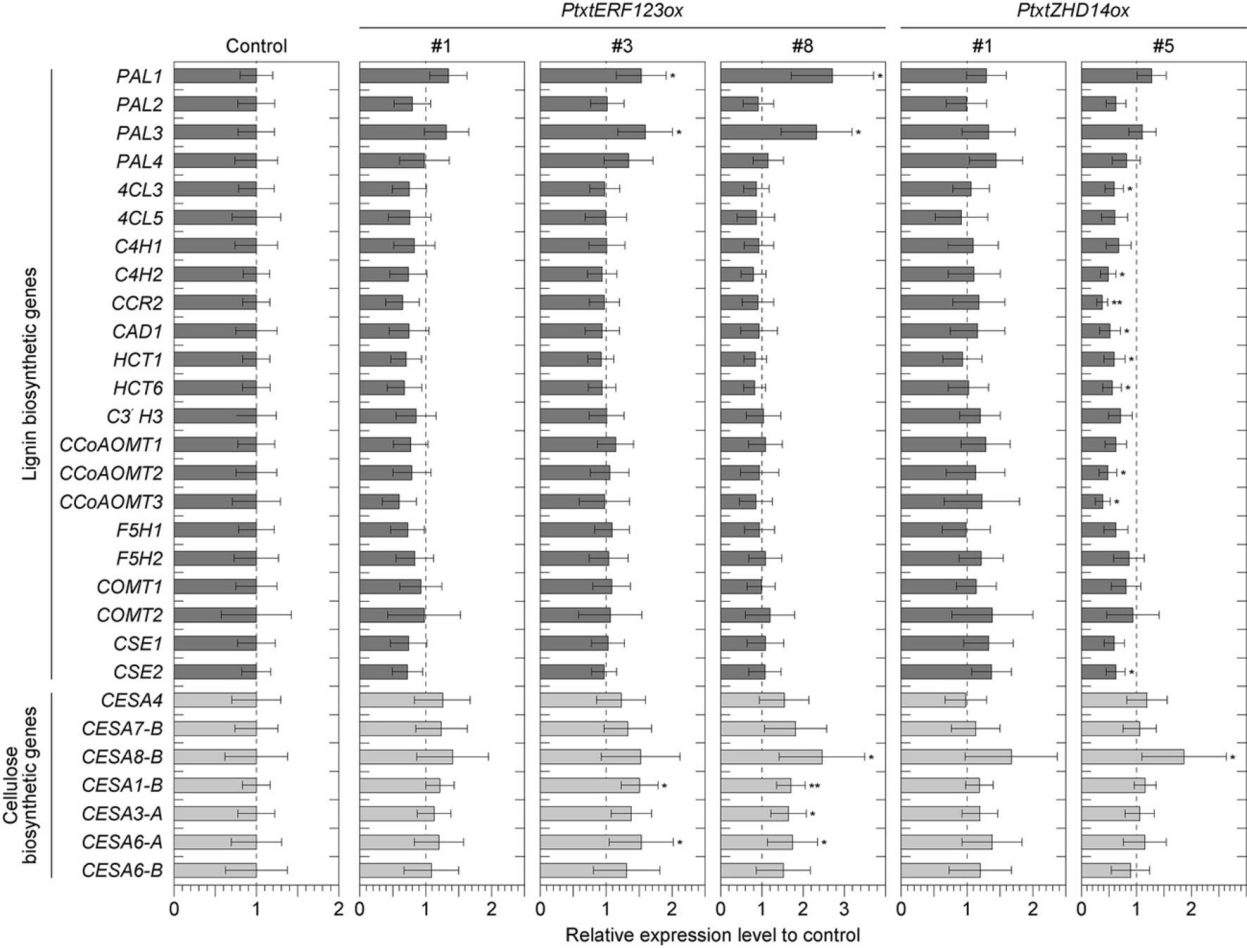
Relative expression level of a total of 29 genes involved in lignin and cellulose biosynthesis in seedling stems of Pt × tERF123ox, and Pt × t ZHD14ox lines grown for one-month in MS culture under aseptic condition, in comparison to the controls (GFP-TUA6). Expressions were measured by real-time PCR by using gene specific primer pairs listed in Table S1, expression levels between samples were normalized by using 18S ribosomal RNA gene expression, and then relative expressions were calculated by using expression levels in control as 1. Data are shown as mean ± standard deviations of biological triplicates. Single and double asterisk(s) indicate *P* value < 0.05 and < 0.01, respectively in Student’s *t*- test when compared with the control plants.

To further investigate the cause of the lignocellulose component alterations in Pt × tERF123ox and Pt × tZHD14ox, we assessed the changes in the expression of lignin and cellulose biosynthetic genes in Pt × tERF123ox and Pt × tZHD14ox. Expression levels of 22 lignin biosynthetic genes, including those encoding phenylalanine ammonium (PAL), 4-coumarate-CoA ligase (4CL), C4H, CCR, CAD, HCT, C3′H, CCoAOMT, F5H, COMT, cafferoyl shikimate esterase (CSE), and also 7 CESA genes involved in cellulose biosynthesis were measured by real time PCR using RNA extracted from the seedling stems as samples (Fig. 4). In case of Pt × tERF123ox lines, compared with control lines, the relative expression of most of the lignin biosynthetic genes tested, except for PAL1 and PAL3, were comparable or lower albeit without statistical significance, which was overall in line with the reduced lignin content (Table 2). In contrast, a series of CESA genes appeared to be apparently upregulated in Pt × tERF123ox. In particular, CESA1-B, CESA3-A, CESA6-A and CESA6-B associated with PCW formation^41^ as well as CESA8-B associated with SCW formation^12,42^ were significantly upregulated (Fig. 4). The fold changes of the CESA expression were in a descending order of line #8 > #3 > #1, which was apparently correlated with the cellulose content and saccharification performance determined earlier (Table 2, Fig. 3). In case of Pt × tZHD14ox line #1, relative expressions of cellulose and lignin biosynthetic genes were comparable or slightly higher in Pt × tZHD14ox lines. On the other hand, in Pt × tZHD14ox line #5, expression levels of several lignin biosynthetic genes including 4CL, C4H, CCR, CAD, HCT, CCoAOMT and CSE were significantly reduced, whereas gene expression of CESA8-B for cellulose biosynthesis was significantly increased, compared to the control lines.

## Discussion

In this study, we successfully generated and screened transgenic hybrid aspen overexpressing 33 TFs with a potential role in regulating xylem cell wall biosynthesis (Table 1; Fig. 1A). Four of the previously uncharacterized TFs (Pt × tERF123, Pt × tZHD14, Pt × tTCL1 and Pt × tWLIM2B) had increased saccharification of seedling tissue on average when overexpressed (Fig. 1B). Among them, all lines of Pt × tERF123ox and Pt × tZHD14ox which showed the excellent saccharification performance at the seedling screening stage were further grown to form mature xylem in the greenhouse. Consequently, most of the tested Pt × tERF123ox and Pt × tZHD14ox lines displayed significantly increased initial glucan hydrolysis rates (glucose release after 3 h-treatment of enzyme cocktail) albeit with less significant enhancement in the total glucan degradation (glucose release after 48 h-treatment of enzyme cocktail) by enzymatic saccharification (Fig. 3). Notably, all the Pt × tERF123ox and Pt × tZHD14ox lines displayed increased xylan degradations (xylose release after 48 h-treatment of enzyme cocktail) with 1.5–2.0 times higher xylose yields compared to the controls. Xylan is thought to be one of the limiting factors in enzymatic hydrolysis of cellulose possibly because xylan physically covers cellulose surface in lignocellulose and thereby hinders the access of cellulolytic enzymes to cellulose substrate^43^. Therefore, easier removal of xylan may have enhanced cellulose hydrolysis of the Pt × tERF123ox and Pt × tZHD14ox cell walls.

Many studies have investigated the relationship between lignocellulose structure and enzymatic saccharification performance of cell wall materials. Major factors that affect cell wall saccharification performance are thought to be glucan contents as well as the recalcitrant lignin content and structure which substantially prevents the access of hydrolytic enzymes to cellulose and hemicellulose substrates and subsequent hydrolysis reactions^44,45^. Indeed, our chemical analysis data showed that Pt × tERF123ox displayed relatively increased glucan and significantly reduced lignin content, albeit with no apparent change in lignin aromatic composition, compared to the control (Table 2). Thus, it is plausible that the improved saccharification performance of Pt × tERF123ox is due to the reduced lignin recalcitrance, besides the reduced xylan barrier. The alteration of cell wall composition in Pt × tERF123ox, i.e., reduced lignin and increased cellulosic glucan levels, could be attributed to the up-regulation of a series of cellulose synthase genes upon overexpression of Pt × tERF123 (Fig. 4). Previously, several *Populus* ERFs were reported to be involved in the formation of tension wood (TW) which typically produce cell walls with reduced lignin and increased cellulose compared to normal wood cell walls^34^. In addition, a member of *ERF* gene family in *A. thaliana*, *AtERF035*, was recently identified as an active regulator of PCW formation; it was demonstrated that overexpression of *AtERF035* substantially enrich pectin and cellulose proportionally over lignin in the cell walls through induction of PCW formation^41^. Intriguingly, *Pt* × *tERF123* was significantly up-regulated during the cell expansion together with PCW-associated enzyme genes (Figure S2), and CESA1-B, CESA3-A and CESA6-A associated with PCW formation were significantly upregulated (Fig. 4). Therefore, it is possible that the altered lignocellulose composition detected in the Pt × tERF123ox cell walls (Table 2) might be associated with cell wall alteration analogous to TW and/or PCW formation(s), although our current histochemical and cell wall chemical analysis data do not entirely corroborate this hypothesis (Fig. 2 and Table 2). Further rigorous analyses on the transcription network associated with Pt × tERF123 as well as cell wall structures of *Pt* × *tERF123*-overexpressing and/or -downregulated transgenic aspens are needed to clarify this aspect.

In contrast to Pt × tERF123ox, the improved saccharification of Pt × tZHD14ox cell walls is unlikely to be associated with compositional change of lignin, xylan and cellulose in the cell walls (Table 2). Besides lignin content and structure, several other factors, such as cellulose crystallinity, hemicellulose modifications, and covalent and non-covalent linkages between lignin and polysaccharides, have been proposed as potential factors that may affect cell wall saccharification performance^46,47^. Therefore, future study will need to focus on further structural analyses on the Pt × tZHD14ox cell walls to investigate these factors. In this context, we note that a homologous gene of *Pt* × *tZHD14* in *A. thaliana* (*AtZHD9/AtHB34*) was expressed in various tissues including inflorescence stems^35,48^, but its function remains unclear. In this study, we observed that cell wall thickness was fluctuated along with the introduced expression level of *Pt* × *tZHD14* (Fig. 2E), and contents of some hemicellulosic sugars in cell walls were also affected in the Pt × tZHD14ox poplar lines (Table 2). Given that *Pt* × *tZHD14* is substantially up-regulated during SCW formation together with SCW biosynthetic genes (Figure S2, Fig. 4), it is plausible that Pt × tZHD14 is involved in xylem development in aspen. Nevertheless, as also noted for Pt × tERF123, further detailed analysis on the transcription network associated with Pt × tZHD14 is needed to elucidate its role in xylem cell wall development in *Populus*.

While we found 4 promising TF-overexpressing hybrid aspens with improved cell wall saccharification performance, the other 29 TF-overexpressing lines showing decreased saccharification performance (Fig. 1B) are also of interest for further investigation of their potential role in xylem development in *Populus*. As previously reported, overexpression of both strong transcriptional repressors and/or activators of SCW are expected to cause a decrease of enzymatic digestibility along with striking loss or accumulation of secondary cell wall in the xylem. For example, overexpression of the strong repressors of SCW formation, *KNAT7* and *MYB199*, were reported to result in remarkably thin xylem *Populus* cell walls^31,32^. Also, in the case of strong activators, overexpression of *MYB003* and *MYB152 in planta* deposited ectopic and/or thick cell walls^28,29^. Overall, our results suggested that only approximately 10% of the selected TF up-regulated during wood formation increased enzymatic saccharification, therefore, the first seedling screening method in this study was effective to find transgenic lines with the improve enzymatic digestibility. In *Arabidopsis*, a similar approach successfully identified several cell-wall-related TF genes that increase cell wall saccharification efficiency^49^.

The cultivation and the application of genetically modified organisms are controversial. Recent technology of genome editing has drawn attention to solve this problem since it will enables modulations of gene expression and function without leaving foreign gene in a cell^50^. For example, gene expression can be enhanced by altering *cis* sequence in the upstream region of associated TF genes, or by silencing negative effectors, and also protein function can be modulated by amino acid substitution. The biomass-recalcitrance-associated TFs identified in this study can be considered as promising targets to improve poplar cell wall properties using such genome editing strategies. Moreover, given that biomass conversion often needs pre-treatment before enzymatic saccharification^1^, future study may further investigate the saccharification performance of the TF-overexpressing aspen lines identified in this study using various pretreatment strategies.

Collectively, our strategy to target and screen an array of TFs up-regulated during wood formation successfully constructed transgenic hybrid aspens with reduced xylem cell wall recalcitrance of xylem cell walls without apparent biomass loss. The observation that all cell wall components were coordinately changed in the transgenic aspens in this study is likely due to our target TFs regulating xylem cell wall formation at a global level, not a single cell wall component. Furthermore, the identified TF genes such as *Pt* × *tERF123* and *Pt* × *tZHD14* are widely distributed in various angiosperms^34,35^, therefore, these may be new molecular breeding targets for the improved enzymatic digestibility of various biomass, especially other woody biomass crops, such as eucalyptus and willow.

## Methods

### Plant materials and growth

Sterile plants of *P. tremula* × *P. tremuloides* (wild-type clone T89) were propagated in half strength Murashige and Skoog (MS) medium (pH 5.7) containing 0.8% (w/v) agar at 25 °C under long-day conditions (18-h light at 200 µmol m^-2^ s^-1^/6-h dark)^42^. Hybrid aspens were transplanted into soil mixture (3:1 fertilized peat moss:vermiculite, v/v) and grown in a greenhouse at 20 °C under long-day conditions. Plants were fertilized once a week with 2000-fold diluted Hyponex 6–10-5 solution (HYPONeX Japan Corp., Ltd., Osaka, Japan). Plant height and stem diameter at 10 cm above the soil was measured weekly.

### Bioinformatics

Gene expression patterns of the selected 33 TFs were investigated in the *Populus* tissue expression data^33^ and the AspWood database (http://aspwood.popgenie.org/aspwood-v3.0/) ^21^. We re-analyzed raw counts of RNA sequencing dataset (GSE81077)^33^ retrieved from NCBI Gene Expression Omnibus. Genes with at least one count-per-million (cpm) in all samples were retained and normalized with trimmed mean of *M*-value (TMM) using edgeR, ver. 3.18.1^51^ in R software, ver.3.3.2^52^. To perform gene clustering analysis, we retrieved primary cell wall- and secondary cell wall-related genes from the genome database of *Populus trichocarpa* (https://phytozome.jgi.doe.gov/). The expression patterns of 33 TFs and cell wall-related genes were analyzed in the AspWood database and visualized by expression heatmap.

### Generation of transgenic hybrid aspens

cDNAs of the target TF coding sequences were isolated from *P. tremula* × *P. tremuloides* or *Populus nigra* as described below, using the primers listed in Table S1. The coding sequences (without stop codon) were cloned into the pENTR/D-TOPO vector (Thermo Fisher Scientific, Waltham, MA, USA), and then transferred to the pGWB560 vector (C-terminal fusion with TagRFP) by the Gateway LR reaction^53^. pGWB560 harboring the NLS-TagRFP was used as a control vector. The constructed binary vectors were introduced into a hybrid aspen, which expresses *soluble-modified RED-SHIFTED GREEN FLUORESCENT PROTEIN* (*smRSGFP*)-tagged *Arabidopsis thaliana ALPHA TUBULIN-6* (*TUA6*) (GFP-TUA6) driven by *Cauliflower Mosaic Virus 35S* (*CaMV35S*) promoter, by *Agrobacterium*-mediated transformation^54,55^. Ectopic expression of *GFP-TUA6* did not cause any observable effects on plant growth or xylem structure, such as the length and width of wood fibers and vessel elements (Figure S3).

### RNA extraction and real-time PCR

To check relative expression level of the transgene (TF-TagRFP) in transgenic hybrid aspens, leaves were harvested from sterile plants grown for one month, frozen in liquid nitrogen, and total RNA was isolated using an RNeasy Plant Mini Kit (Qiagen, Hilden, Germany) with in-column DNase I digestion. First-strand cDNA was synthesized using a High Capacity RNA-to-cDNA Kit (Thermo Fisher Scientific). Real-time PCR was performed using a StepOnePlus Real-Time PCR System with Power SYBRGreen PCR Master Mix (Thermo Fisher Scientific). Expression of the introduced genes was measured using TagRFP region-amplified primers (Table S1). A primer pair designed to target *Pt* × *tUBQ* was used as an internal standard (Table S1). No amplicon was detected in the control T89 and GFP-TUA6 plants when using the TagRFP primer pair in real-time PCR analysis.

To measure relative expression level of genes involved in lignin and cellulose biosynthesis in Pt × tERF123ox, Pt × t ZHD14ox, the seedling stems were harvested from sterile plants grown for one month and stored in liquid nitrogen. RNA extraction and cDNA synthesis were conducted as described above, and expressions were measured by real-time PCR by using gene specific primer pairs for 22 lignin biosynthetic genes and 7 cellulose biosynthetic genes listed in Table S1. A primer pair designed to 18S ribosomal RNA was used as an internal standard (Table S1). Relative expressions were calculated by using average of expression levels of control lines as a standard (relative expression level of control = 1.0).

### Enzymatic saccharification of transgenic hybrid aspens

For transgenic hybrid aspen grown in aseptic conditions, stem tissues were lyophilized and ground into fine powder using a crusher (µT-12, TAITEC Corporation, Saitama, Japan) at 2000 rpm for 30 s, repeated three times. For transgenic aspen grown in the greenhouse, harvested plant tissues were lyophilized and debarked, and the xylem tissues were ground into fine powder as described above. The saccharification assay was modified from the NREL protocol^56^. Briefly, powdered sample (10 mg) was mixed with 1 mL of 1 mg/mL enzyme cocktail in 50 mM sodium acetate buffer (pH 5.0) with 0.1% ampicillin. The mixture was incubated at 50 °C for 48 h with inversion mixing by rotator at approximately 30 rpm (RT-50, TAITEC). The enzyme cocktail included Cellulase from *Trichoderma reesei* ATCC 26921 (Merck KGaA, Darmastat, Germany) and Cellobiase from *Aspergillus niger* (Novozyme 188, Merck) with a protein concentration ratio of 4:1. The strain of *Trichoderma reesei* ATCC26921 is a well-known enhanced cellulase-producing mutant as QM9414 derived from a wild-type strain of QM6a^57^. The enzyme mixture includes cellulolytic enzymes such as cellobiohydrolase, endoglucanase and beta-1,4-glucosidase as well as hemicellulolytic enzymes such as mannanase and xylanase, xylosidase, arabinofuranosidase, and other enzymes to release glucose and xylose from cellulosic biomass^58^. To accurately measure released glucose, beta-1,4-glucosidase (cellobiase) was added to the cellulase mixture. Additional xylanase loading in the enzyme cocktail did not increase the glucose release from poplar seeding stem samples. The amount of released glucose and xylose in the supernatant was measured using the LABASSAY GLUCOSE (FUJIFILM Wako Pure Chemical Corporation, Osaka, Japan) and D-Xylose assay kits (https://www.megazyme.com/documents/Booklet/K-XYLOSE_DATA.pdf, Megazyme Ltd., Wicklow, Ireland), respectively.

### Anatomical observation of hybrid aspen xylem tissues

Stem samples were harvested from the 20th internode and fixed in FAA solution (50% ethanol, 10% formaldehyde, and 5% acetic acid). Trimmed stem segments were dehydrated in a graded ethanol series (50%, 60%, 80%, 90%, and 100%), and were embedded in LR White Hard resin (TAAB, Aldermaston, UK) with 5% PEG400. Cross Sections (2-µm thick) were cut using a rotary microtome (RX-860; Yamato Kohki Industrial, Saitama, Japan) and then stained with a 0.5% (w/v) toluidine blue solution. Sections were imaged using a Leica DMi8 microscope with a Leica DFC7000T microscope camera (Leica Microsystems, Wetzlar, Germany). Cell wall thickness and cell size of wood fiber cells were measured using the ImageJ software (n = 100 for each plant)^59,60^.

### Chemical analysis of hybrid aspen xylem tissues

The ground xylem powder was extracted sequentially by water and 80% (v/v) ethanol to generate extractive-free cell wall residues^61^. The thioglycolic acid lignin content assay^38^ and thioacidolysis lignin composition analysis^38,39^ were carried out as described previously. For determination of the non-cellulosic cell wall polysaccharide composition, the extractive-free cell wall residues were first hydrolyzed by trifluoroacetic acid. The released monosaccharides were converted into alditol acetates and subjected to analysis by gas chromatography–mass spectrometry with inositol acetate as an internal standard^39,40^. To determine crystalline glucan content, the remaining residues were treated with Updegraff reagent^62^, followed by complete hydrolysis using 72% (v/v) sulfuric acid^63^. Glucose released was quantified by the Glc CII test kit (FUJIFILM Wako Pure Chemical Corporation).

## Supplementary Information

Supplementary Information

## Abbreviations

4CL: 4-Coumarate-CoA ligase
C3′H: *p*-Coumarate 3-hydroxylase
C4H: Cinnamate 4-hydroxylase
CAD: Cinnamyl alcohol dehydrogenase
CaMV35S: Cauliflower Mosaic Virus 35S
CCoAOMT: Caffeoyl-CoA *O*-methyltransferase
CCR: Cinnamoyl-CoA reductase
CESA: Cellulose synthase
COMT: Caffeic acid *O*-methyltransferases
CSE: Cafferoyl shikimate esterase
CSL: Cellulose synthase-like
ERF: Ethylene response factor
F5H: Ferulate 5-hydroxylase
FRA: Fragile fiber
G: Guaiacyl
GALS: Galactosyltransferase
GATL: Galacturonosyltransferase-like
GAUT: Galacturonosyltransferase
GFP: Green fluorescent protein
GT: Glycosyltransferase
H: *p*-Hydroxyphenyl
HCT: *p*-Hydroxycinnamoyl-CoA:quinate/shikimate *p*-hydroxycinnamoyltransferase
IRX: Irregular xylem
MS: Murashige and Skoog
NAC: No apical meristem/Arabidopsis transcription activation factor/cup-shaped cotyledon
NLS: Nuclear localization sequence
NST/SND: NAC secondary wall thickening promoting factor/secondary wall-associated NAC domain
PAL: Phenylalanine ammonium lyase
PCW: Primary cell wall
Pt × t: *Populus tremula* × *Populus tremuloides*
S: Syringyl
SCW: Secondary cell wall
SMB: Sombrero
TCL: Trichomeless
TF: Transcriptional factor
TW: Tension wood
TUA: Alpha tubulin
VND: Vascular-related NAC domain
VNS: VND, NST/SND, and SMB related
WLIM: Widely expressed LIN-11, Isl1 and MEC-3 (LIM)-structural domain protein
XXT: Xylosyltransferase
ZHD: Zinc finger homeodomain

## References

[1] Tursi A (2019). A review on biomass: Importance, chemistry, classification, and conversion. Biofuel Res. J..

[2] Himmel ME (2007). Biomass recalcitrance: Engineering plants and enzymes for biofuels production. Science.

[3] Porth I, El-Kassaby YA (2015). Using *Populus* as a lignocellulosic feedstock for bioethanol. Biotechnol. J..

[4] Donaldson LA, Paul Knox J (2012). Localization of cell wall polysaccharides in normal and compression wood of radiata pine: Relationships with lignification and microfibril orientation. Plant Physiol..

[5] Müller M, Hori R, Itoh T, Sugiyama J (2002). X-ray microbeam and electron diffraction experiments on developing xylem cell walls. Biomacromol.

[6] Ye ZH, Zhong R (2015). Molecular control of wood formation in trees. J. Exp. Bot..

[7] Nakano Y, Yamaguchi M, Endo H, Rejab NA, Ohtani M (2015). NAC-MYB-based transcriptional regulation of secondary cell wall biosynthesis in land plants. Front. Plant Sci..

[8] Zhang J, Xie M, Tuskan GA, Muchero W, Chen JG (2018). Recent advances in the transcriptional regulation of secondary cell wall biosynthesis in the woody plants. Front. Plant Sci..

[9] Kubo M (2005). Transcription switches for protoxylem and metaxylem vessel formation. Genes Dev..

[10] Mitsuda N (2007). NAC transcription factors, NST1 and NST3, are key regulators of the formation of secondary walls in woody tissues of *Arabidopsis*. Plant Cell.

[11] Zhong R, Demura T, Ye Z-H (2006). SND1, a NAC domain transcription factor, is a key regulator of secondary wall synthesis in fibers of *Arabidopsis*. Plant Cell.

[12] Ohtani M (2011). A NAC domain protein family contributing to the regulation of wood formation in poplar. Plant J..

[13] Hu R (2010). Comprehensive analysis of NAC domain transcription factor gene family in *Vitis vinifera*. BMC Plant Biol..

[14] Takata N (2019). *Populus* NST/SND orthologs are key regulators of secondary cell wall formation in wood fibers, phloem fibers and xylem ray parenchyma cells. Tree Physiol..

[15] Li Q (2012). Splice variant of the SND1 transcription factor is a dominant negative of SND1 members and their regulation in *Populus trichocarpa*. Proc. Natl. Acad. Sci. USA..

[16] Zhong R, Lee C, Ye Z-H (2010). Functional characterization of poplar wood-associated NAC domain transcription factors. Plant Physiol..

[17] Zhong R, Mccarthy RL, Lee C, Ye ZH (2011). Dissection of the transcriptional program regulating secondary wall biosynthesis during wood formation in poplar. Plant Physiol..

[18] Zhao Y, Sun J, Xu P, Zhang R, Li L (2014). Intron-mediated alternative splicing of WOOD-ASSOCIATED NAC TRANSCRIPTION FACTOR1B regulates cell wall thickening during fiber development in *Populus* species. Plant Physiol..

[19] Courtois-Moreau CL (2009). A unique program for cell death in xylem fibers of *Populus* stem. Plant J..

[20] Hertzberg M (2001). A transcriptional roadmap to wood formation. Proc. Natl. Acad. Sci. U.S.A..

[21] Sundell D (2017). AspWood: High-spatial-resolution transcriptome profiles reveal uncharacterized modularity of wood formation in *Populus tremula*. Plant Cell.

[22] Lin YC (2013). SND1 transcription factor-directed quantitative functional hierarchical genetic regulatory network in wood formation in *Populus trichocarpa*. Plant Cell.

[23] Chen H (2019). Hierarchical transcription factor and chromatin binding network for wood formation in *Populus trichocarpa*. Plant Cell.

[24] Gui J (2020). Fibre-specific regulation of lignin biosynthesis improves biomass quality in *Populus*. New Phytol..

[25] Cho JS (2019). Wood forming tissue-specific bicistronic expression of *PdGA20ox1* and *PtrMYB221* improves both the quality and quantity of woody biomass production in a hybrid poplar. Plant Biotechnol. J..

[26] Brandon AG, Scheller HV (2020). Engineering of bioenergy crops: Dominant genetic approaches to improve polysaccharide properties and composition in biomass. Front. Plant Sci..

[27] Wang JP (2018). Improving wood properties for wood utilization through multi-omics integration in lignin biosynthesis. Nat. Commun..

[28] McCarthy RL (2010). The poplar MYB transcription factors, PtrMYB3 and PtrMYB20, are involved in the regulation of secondary wall biosynthesis. Plant Cell Physiol..

[29] Li C (2014). A poplar R2R3-MYB transcription factor, PtrMYB152, is involved in regulation of lignin biosynthesis during secondary cell wall formation. Plant Cell. Tissue Organ Cult..

[30] Wang S (2014). Regulation of secondary cell wall biosynthesis by poplar R2R3 MYB transcription factor PtrMYB152 in *Arabidopsis*. Sci. Rep..

[31] Tang X (2020). Dual regulation of xylem formation by an auxin-mediated PaC3H17-PaMYB199 module in *Populus*. New Phytol..

[32] Li E (2012). The Class II KNOX gene KNAT7 negatively regulates secondary wall formation in Arabidopsis and is functionally conserved in *Populus*. New Phytol..

[33] Shi R (2017). Tissue and cell-type co-expression networks of transcription factors and wood component genes in *Populus trichocarpa*. Planta.

[34] Vahala J (2013). A genome-wide screen for ethylene-induced Ethylene Response Factors (ERFs) in hybrid aspen stem identifies *ERF* genes that modify stem growth and wood properties. New Phytol..

[35] Hu W, Depamphilis CW, Ma H (2008). Phylogenetic analysis of the plant-specific zinc finger-homeobox and mini zinc finger gene families. J. Integr. Plant Biol..

[36] Zhou L (2014). Control of trichome formation in *Arabidopsis* by poplar single-repeat R3 MYB transcription factors. Front. Plant Sci..

[37] Arnaud D, Déjardin A, Leplé JC, Lesage-Descauses MC, Pilate G (2007). Genome-wide analysis of LIM gene family in *Populus trichocarpa, Arabidopsis thaliana*, and *Oryza sativa*. DNA Res..

[38] Suzuki S (2009). High-throughput determination of thioglycolic acid lignin from rice. Plant Biotechnol..

[39] Lam PY (2017). Disrupting flavone synthase II alters lignin and improves biomass digestibility. Plant Physiol..

[40] Chen F, Tobimatsu Y, Havkin-Frenkel D, Dixon RA, Ralph J (2012). A polymer of caffeyl alcohol in plant seeds. Proc. Natl. Acad. Sci. U.S.A..

[41] Sakamoto S (2018). Complete substitution of a secondary cell wall with a primary cell wall in *Arabidopsis*. Nat. Plants.

[42] Takata N, Taniguchi T (2015). Expression divergence of cellulose synthase (CesA) genes after a recent whole genome duplication event in *Populus*. Planta.

[43] Penttilä PA (2013). Xylan as limiting factor in enzymatic hydrolysis of nanocellulose. Bioresour. Technol..

[44] Chen F, Dixon RA (2007). Lignin modification improves fermentable sugar yields for biofuel production. Nat. Biotechnol..

[45] Li M, Pu Y, Ragauskas AJ (2016). Current understanding of the correlation of lignin structure with biomass recalcitrance. Front. Chem..

[46] Zheng M (2014). Protein expression changes during cotton fiber elongation in response to drought stress and recovery. Proteomics.

[47] Marriott PE, Gómez LD, Mcqueen-Mason SJ (2016). Unlocking the potential of lignocellulosic biomass through plant science. New Phytol..

[48] Tan QKG, Irish VF (2006). The Arabidopsis zinc finger-homeodomain genes encode proteins with unique biochemical properties that are coordinately expressed during floral development. Plant Physiol..

[49] Ohtani M (2017). Identification of novel factors that increase enzymatic saccharification efficiency in *Arabidopsis* wood cells. Plant Biotechnol..

[50] Bewg WP, Ci D, Tsai CJ (2018). Genome editing in trees: From multiple repair pathways to long-term stability. Front. Plant Sci..

[51] Robinson MD, McCarthy DJ, Smyth GK (2010). edgeR: A bioconductor package for differential expression analysis of digital gene expression data. Bioinformatics.

[52] R Core Team. R: A language and environment for statistical computing. *R Found. Stat. Comput. Vienna, Austria.* Available at https://www.r-project.org/. (2018).

[53] Nakagawa T (2007). Improved gateway binary vectors: High-performance vectors for creation of fusion constructs in transgenic analysis of plants. Biosci. Biotechnol. Biochem..

[54] Eriksson ME, Israelsson M, Olsson O, Moritz T (2000). Increased gibberellin biosynthesis in transgenic trees promotes growth, biomass production and xylem fiber length. Nat. Biotechnol..

[55] Ueda K, Matsuyama T, Hashimoto T (1998). Visualization of microtubules in living cells of transgenic *Arabidopsis thaliana*. Protoplasma.

[56] Resch, M.G., Baker, J.O. & Nrel, S.R.D. Low solids enzymatic saccharification of lignocellulosic biomass. *NREL Lab. Anal. Proced.* (2015).

[57] Bailey MJ, Nevalainen KMH (1981). Induction, isolation and testing of stable *Trichoderma reesei* mutants with improved production of solubilizing cellulase. Enzyme Microb. Technol..

[58] Adav SS (2011). Proteomic analysis of pH and strains dependent protein secretion of *Trichoderma reesei*. J. Proteome Res..

[59] Dougherty R, Kunzelmann K-H (2007). Computing local thickness of 3D structures with ImageJ. Microsc. Microanal..

[60] Nuoendagula (2018). Change in lignin structure, but not in lignin content, in transgenic poplar overexpressing the rice master regulator of secondary cell wall biosynthesis. Physiol. Plant..

[61] Mansfield SD, Kim H, Lu F, Ralph J (2012). Whole plant cell wall characterization using solution-state 2D NMR. Nat. Protoc..

[62] Updegraff DM (1969). Semimicro determination of cellulose inbiological materials. Anal. Biochem..

[63] Hattori T (2012). Rapid analysis of transgenic rice straw using near-infrared spectroscopy. Plant Biotechnol..

