# Supplementary material for: Identifying transcription factors that reduce wood recalcitrance and improve enzymatic degradation of xylem cell wall in Populus
Source: Sci Rep. 2020 Dec 16;10:22043. doi: 10.1038/s41598-020-78781-6 (PMC7744511; doi:10.1038/s41598-020-78781-6)
Supplement: Supplementary file 1 — Supplementary Information [file 41598_2020_78781_MOESM1_ESM.pdf]

Supplementary information for

Identifying transcription factors that reduce wood recalcitrance and improve enzymatic degradation of xylem cell wall in poplar

Chiaki Hori,[1†*] Naoki Takata,[2†] Pui Ying Lam,[3] Yuki Tobimatsu,[3]

Soichiro Nagano,[4] Jenny C. Mortimer,[5] Dan Cullen,[6]

[1]Research Faculty of Engineering, Hokkaido University, Sapporo 060-8628, Japan

[2]Forest Bio-Research Center, Forestry and Forest Products Research Institute, Forest Research and Management Organization, Hitachi, Ibaraki 319-1301, Japan

[3]Research Institute for Sustainable Humanosphere, Kyoto University, Uji, Kyoto, 611-0011, Japan

[4]Forest Tree Breeding Center, Forestry and Forest Products Research Institute, Forest Research and Management Organization, Hitachi, Ibaraki 319-1301, Japan

[5]Joint BioEnergy Institute, Environmental Genomics and Systems Biology Division, Lawrence Berkeley National Laboratory, Berkeley, CA 94720, USA

[6]U. S. Department of Agriculture, Forest Products Laboratory, Madison, WI 53726, USA

[†]These authors equally contributed to this study.

*Corresponding author

Chiaki Hori, PhD.

Assistant Professor, Hokkaido University

Email, chori@eng.hokudai.ac.jp; Phone, +81-11-706-6612; Fax, +81-11-706-6612

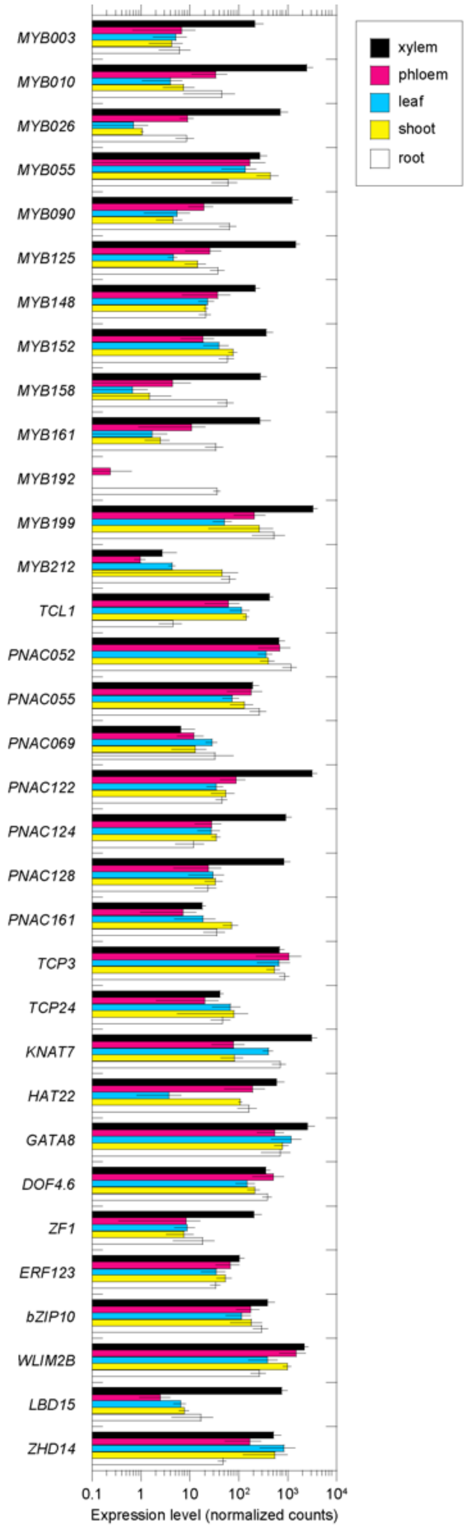

Figure S1. Expression pattern of target TFs in xylem, phloem, leaf, shoot, and root tissues in poplar. RNA sequencing dataset (GSE81077)[1] was retrieved and re-analyzed.

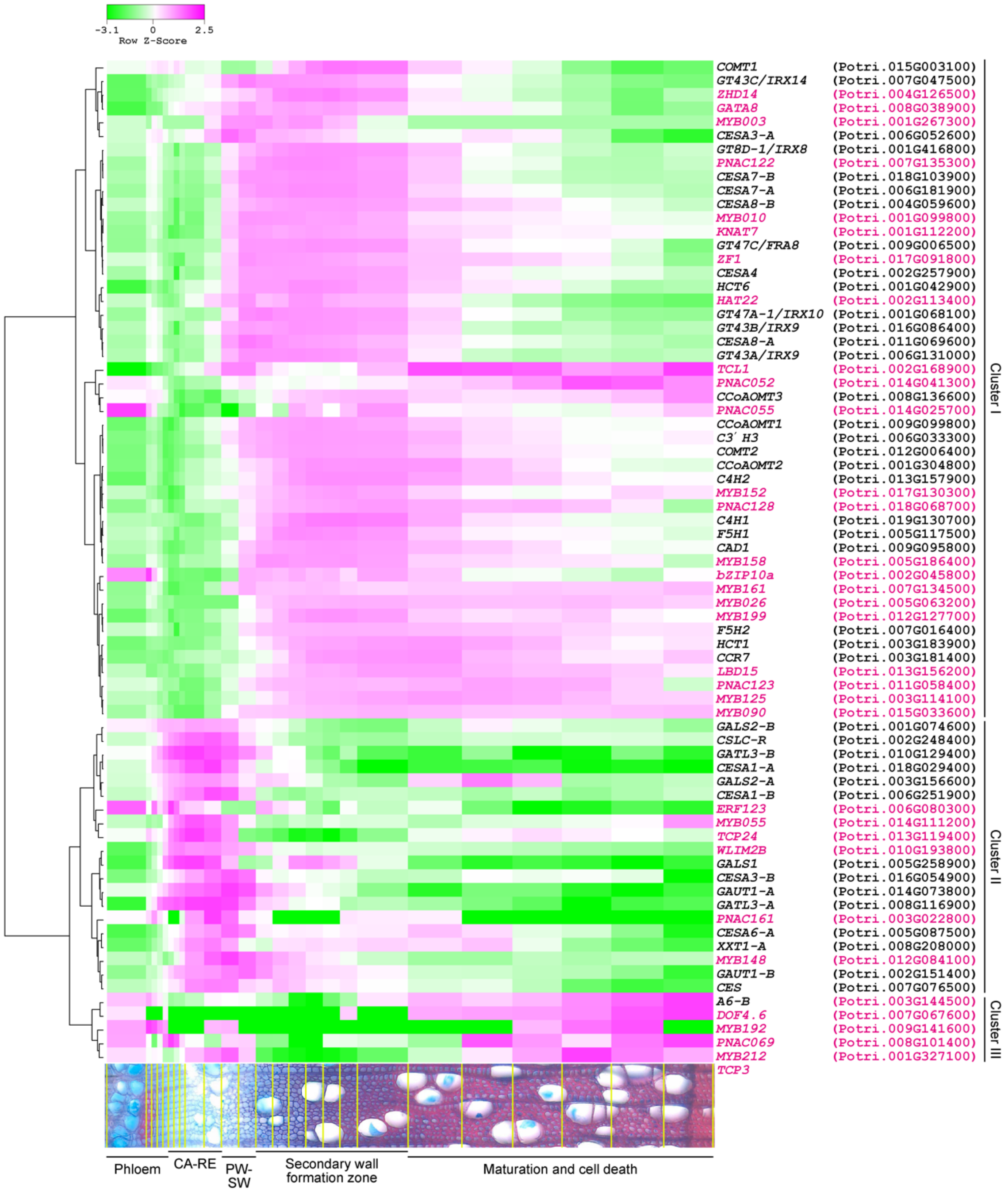

Figure S2. Gene expression patterns of 33 target TFs during xylem formation (gene names in magenta) together with 40 biosynthetic enzymes of cellulose, hemicellulose and lignin (gene names in black), based on the information deposited in the AspWood database (http://aspwood.popgenie.org/aspwood-v3.0/)[2]. Classification of stem tissue was referred to Kumar et al (2019)[3]. CA-RE; cambium and radial expansion zone. PW-SW; Primary to secondary wall transition.

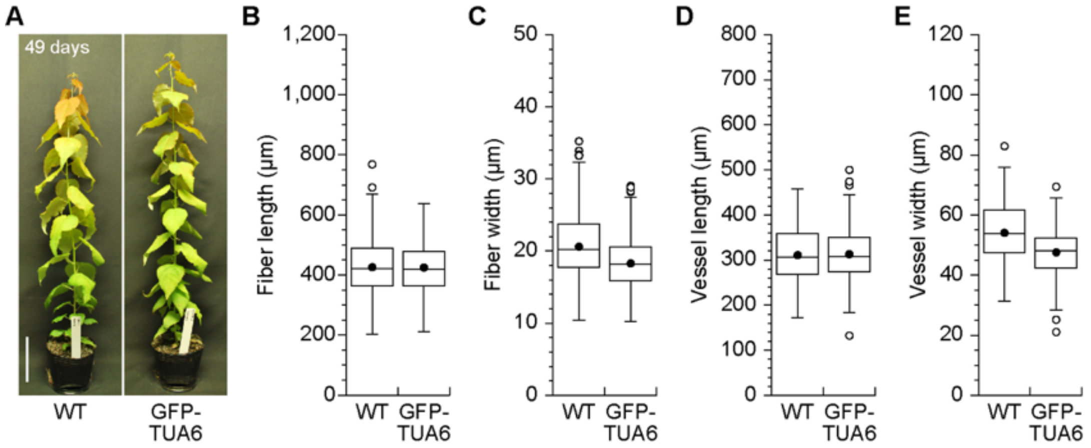

Figure S3. Transgenic hybrid aspen overexpressing GFP-TUA6 and wild-type (WT) plants. Phenotypes of 49-days-old WT and GFP-TUA6 (A). Scale bar = 10 cm. Xylem fiber lengths (B) and widths (C), and vessel element lengths (D) and widths (E). Stem samples were macerated in a solution of 30% hydrogen peroxide and glacial acetic acid (1:1) at 95°C for five hours. Xylem cells were imaged using a Leica DMR microscope (Leica Microsystems, Wetzlar, Germany). Length and width were measured using ImageJ software. Six hundred fiber cells and 195 vessel cells were estimated from three trees per genotype.

Table S1. Oligonucleotides used in this study.

Primer Name	Sequence (5'-3')	Target Gene	Experiment
MYB003.TOPO.for	CACCATGATGAGGAAGCCGGAT	*Pt×tMYB003*	Cloning of target gene
MYB003.TOPO.rev	TACTACTTGGAAATCAAGTAAAGG	*Pt×tMYB003*	Cloning of target gene
MYB010.TOPO.for	CACCATGATGGGTCACCATTCTTGCTGC	*Pt×tMYB010*	Cloning of target gene
MYB010.TOPO.rev	TAGAGATGAAGGGAAAGAAGAGA	*Pt×tMYB010*	Cloning of target gene
MYB026.TOPO.for	CACCATGATGGAAGATTTTAGGGGTAGC	*Pt×tMYB026*	Cloning of target gene
MYB026.TOPO.rev	AGAGATCCCCACACCAAGAAAG	*Pt×tMYB026*	Cloning of target gene
MYB055.TOPO.for	CACCATGGGGAGGCACTCTTGTTGCT	*PniMYB055*	Cloning of target gene
MYB055.TOPO.rev	AATATATCCATATGATGCTGTAAG	*PniMYB055*	Cloning of target gene
MYB090.TOPO.for	CACCATGATGTGTACTAGAGGCC	*Pt×tMYB090*	Cloning of target gene
MYB090.TOPO.rev	AGCTGAGAAAAAATCAATGAATG	*Pt×tMYB090*	Cloning of target gene
MYB125.TOPO.for	CACCATGATGGGAAGGCAACCTTGCTGTG	*Pt×tMYB125*	Cloning of target gene
MYB125.TOPO.rev	GCGATTGAGTTCCATCTCTA	*Pt×tMYB125*	Cloning of target gene
MYB148.TOPO.for	CACCATGGGACGACATTCTTGTTGTTTG	*Pt×tMYB148*	Cloning of target gene
MYB148.TOPO.rev	TATCTGGTGGAAGCCATCGA	*Pt×tMYB148*	Cloning of target gene
MYB152.TOPO.for	CACCATGGGAAGGCAACCATGT	*PniMYB152*	Cloning of target gene
MYB152.TOPO.rev	CAAGAGCCCATTTGTCCAAG	*PniMYB152*	Cloning of target gene
MYB158.TOPO.for	CACCATGATGGAGGAATCTTTGGCTGGT	*Pt×tMYB158*	Cloning of target gene
MYB158.TOPO.rev	AGAAGATATACCCACACCAAG	*Pt×tMYB158*	Cloning of target gene
MYB161.TOPO.for	CACCATGATGTCTAGCAGAGGCCA	*Pt×tMYB161*	Cloning of target gene
MYB161.TOPO.rev	CAATCCACTAACTTGGTTTCCAGC	*Pt×tMYB161*	Cloning of target gene
MYB192.TOPO.for	CACCATGATGGGAAAAGGGAGAGCAC	*Pt×tMYB192*	Cloning of target gene
MYB192.TOPO.rev	TAAAGAAGGCCACAAATGAA	*Pt×tMYB192*	Cloning of target gene
MYB199.TOPO.for	CACCATGATGGGTAGACAACCTTGTTG	*Pt×tMYB199*	Cloning of target gene
MYB199.TOPO.rev	ATGCTTGCCACCCATGTCTA	*Pt×tMYB199*	Cloning of target gene
MYB212.TOPO.for	CACCATGATGGATGTTAAAGGCAACAGCAAC	*Pt×tMYB212*	Cloning of target gene
MYB212.TOPO.rev	CAAGTTGTTTAGCTGCTGCTGTAAGAAC	*Pt×tMYB212*	Cloning of target gene
TCL1.TOPO.for	CACCATGGATAGACGTCGCAAGAAGCG	*Pt×tTCL1*	Cloning of target gene
TCL1.TOPO.rev	AGAGGTATTCGGAATTACATCTCT	*Pt×tTCL1*	Cloning of target gene
PNAC052.TOPO.for	CACCATGCTCTTGCGAGCAATTCCAGGTG	*Pt×tPNAC052*	Cloning of target gene

PNAC052.TOPO.rev	GAAGAAGAAGTAAAATGGAACGAAGC	*Pt×tPNAC052*	Cloning of target gene
PNAC055.TOPO.for	CACCATGGAAGAGAGCAACACAAACCCCAAAAC	*Pt×tPNAC055*	Cloning of target gene
PNAC055.TOPO.rev	ATCCACAAGATCATCCAGCTCTATAAAATC	*Pt×tPNAC055*	Cloning of target gene
PNAC069.TOPO.for	CACCATGGATGAAATGCCATTACAGCTTGATC	*Pt×tPNAC069*	Cloning of target gene
PNAC069.TOPO.rev	GAAATTCAAAATGTCTGCATAATGGGCAA	*Pt×tPNAC069*	Cloning of target gene
PNAC122.TOPO.for	CACCATGACTTGGTGCAATGACTG	*PniPNAC122*	Cloning of target gene
PNAC122.TOPO.rev	AGGGATACGAGAAGATCC	*PniPNAC122*	Cloning of target gene
PNAC124.TOPO.for	CACCATGATGACATGGTGCAATAATAATTC	*Pt×tPNAC124*	Cloning of target gene
PNAC124.TOPO.rev	TTTTCTCTGAAGCTTTCCTTTG	*Pt×tPNAC124*	Cloning of target gene
PNAC128.TOPO.for	CACCATGAGTACTAAGTGTAATATGGC	*Pt×tPNAC128*	Cloning of target gene
PNAC128.TOPO.rev	CCCATGATGATCCTGGTT	*Pt×tPNAC128*	Cloning of target gene
PNAC161.TOPO.for	CACCATGGCTGCCAATCTTCCTCCT	*Pt×tPNAC161*	Cloning of target gene
PNAC161.TOPO.rev	ATTTGGCAAACTTATTTCATCAAG	*Pt×tPNAC161*	Cloning of target gene
TCP3.TOPO.for	CACCATGGATCCCAAGGGCTCTAACTCAA	*Pt×tTCP3*	Cloning of target gene
TCP3.TOPO.rev	CTGCCCTGATCCTTGTGAATCATCT	*Pt×tTCP3*	Cloning of target gene
TCP24.TOPO.for	CACCATGAAGAACAACGGAGAAATTATTCAGG	*Pt×tTCP24*	Cloning of target gene
TCP24.TOPO.rev	ATTCTGAGAATTTGGAGAAGAGTGTGGC	*Pt×tTCP24*	Cloning of target gene
KNAT7.TOPO.for	CACCATGCAAGAACCAAACTTGGGC	*PniKNAT7*	Cloning of target gene
KNAT7.TOPO.rev	CCTTTTGCGCTTGGACTTCA	*PniKNAT7*	Cloning of target gene
HAT22.TOPO.for	CACCATGATGGGTTTTGGAACTACTGATGATC	*Pt×tHAT22*	Cloning of target gene
HAT22.TOPO.rev	GCAAGCTGCGGATGAGTGGGTATC	*Pt×tHAT22*	Cloning of target gene
GATA8.TOPO.for	CACCATGATTGGACAGACAAATACTACTAGTAAT	*PniGATA8*	Cloning of target gene
GATA8.TOPO.rev	TATGTAATCCATTGCAGGGTTGCTC	*PniGATA8*	Cloning of target gene
DOF4.6.TOPO.for	CACCATGGATACTGCTCAGTGGCCACAG	*Pt×tDOF4.6*	Cloning of target gene
DOF4.6.TOPO.rev	CCATGATCCTCCACCTAACATT	*Pt×tDOF4.6*	Cloning of target gene
ZF1.TOPO.for	CACCATGAAGAAGAATCAAGAAAAGGTGCA	*PniZF1*	Cloning of target gene
ZF1.TOPO.rev	GTTGGCTATAAGACCCACCAGTG	*PniZF1*	Cloning of target gene
ERF123.TOPO.for	CACCATGGATCCCTGTCAGTCCTCTGGTC	*Pt×tERF123*	Cloning of target gene
ERF123.TOPO.rev	GAGGGAGTCTATCCATTCTATGTCCAG	*Pt×tERF123*	Cloning of target gene
bZIP10.TOPO.for	CACCATGAATAGTGTCTTCTCAGTGGACGA	*Pt×tbZIP10*	Cloning of target gene
bZIP10.TOPO.rev	TTTCTTTTTGGCATCCCTCGCAACC	*Pt×tbZIP10*	Cloning of target gene
WLIM2B.TOPO.for	CACCATGTCGTTTACTGGTACCCAACAGAA	*Pt×tWLIM2B*	Cloning of target gene

WLIM2B.TOPO.rev	AGCCTCTGGCACAGAAGCCGCTG	*Pt×tWLIM2B*	Cloning of target gene
LBD15.TOPO.for	CACCATGTCCAGAGACAGGGAGAGA	*Pt×tLBD15*	Cloning of target gene
LBD15.TOPO.rev	ATCAAAATATGGGACATTATTATC	*Pt×tLBD15*	Cloning of target gene
ZHD14.TOPO.for	CACCATGGACATAACCCCAGCAACAGCAAC	*Pt×tZHD14*	Cloning of target gene
ZHD14.TOPO.rev	AGAAGATGAAGAAGAACCATTAGTCC	*Pt×tZHD14*	Cloning of target gene
NLS-TagRFP.TOPO.for	CACCATGCCTAAGAAGAAGCGTAAGGTTGGAGGAGTGTCTAAGGGCGAAG	*TagRFP*	Cloning of TagRFP
TagRFP.TOPO.rev	ATTAAGTTTGTGCCCCAGTTTG	*TagRFP*	Cloning of TagRFP
PtUBQ.for	GGTTGATTTTTGCTGGGAAGC	*Pt×tUBQ*	Real-time PCR
PtUBQ.rev	GATCTTGGCCTTCACGTTGT	*Pt×tUBQ*	Real-time PCR
TagRFP.for	CAGGACGGCTGCCTCATCT	*TagRFP*	Real-time PCR
TagRFP.rev	GCCGTTGGATGGGAAGTTC	*TagRFP*	Real-time PCR
Ptt18S.RT.for	TCAACTTTCGATGGTAGGATAGTG	*Pt×t18S*	Real-time PCR
Ptt18S.RT.rev	CCGTGTCAGGATTGGGTAATTT	*Pt×t18S*	Real-time PCR
PAL1.RT.for	CGTGAAGATGTCTTTGCTTACGCC	*Pt×tPAL1*	Real-time PCR
PAL1.RT.rev	GCGTTCTTCTCATTCTCTCCATTTG	*Pt×tPAL1*	Real-time PCR
PAL2.RT.for	GCTGTGGGACCCAATGGAGAAA	*Pt×tPAL2*	Real-time PCR
PAL2.RT.rev	GAACCAACAGCAGTACCATTTACA	*Pt×tPAL2*	Real-time PCR
PAL3.RT.for	CGTGAATATGTCTTTGCCTATGTGG	*Pt×tPAL3*	Real-time PCR
PAL3.RT.rev	TCCATTTTCCAACGCATGGTCAAC	*Pt×tPAL3*	Real-time PCR
PAL4.RT.for	GCAATTGGACCCAATGGAGAGC	*Pt×tPAL4*	Real-time PCR
PAL4.RT.rev	CAGAACCCACTGCAGTACTATTTACT	*Pt×tPAL4*	Real-time PCR
C4H2.RT.for	AAACCCTGAGGCTGCAACCAA	*Pt×tC4H1*	Real-time PCR
C4H2.RT.rev	CTCCTCTCACCATTCAAAGCCTTG	*Pt×tC4H1*	Real-time PCR
C4H1.RT.for	AAACCCCGAGGCTGCAACTCAT	*Pt×tC4H2*	Real-time PCR
C4H1.RT.rev	GCTCCTCTCACCATTCAAAGCCTTA	*Pt×tC4H2*	Real-time PCR
4CL3.RT.for	CACACAGAGGTGCCATGATC	*Pt×t4CL3*	Real-time PCR
4CL3.RT.rev	GCCTGTGTTATCAGAAGCTTTGCTC	*Pt×t4CL3*	Real-time PCR
4CL5.RT.for	CACACAGGGGTGCCATTACT	*Pt×t4CL5*	Real-time PCR
4CL5.RT.rev	GCCTGTGTTATCAAAAGCTTTGCTT	*Pt×t4CL5*	Real-time PCR
CSE1.RT.for	CCATGAGAGAAATCGCCAGAGCC	*Pt×tCSE1*	Real-time PCR
CSE1.RT.rev	ACGTCACCCCATCGGCGGTC	*Pt×tCSE1*	Real-time PCR
CSE2.RT.for	CCATGAGGGAAATTGCTAGGATG	*Pt×tCSE2*	Real-time PCR

CSE2.RT.rev	CATGCCACCCCATCAGCCGTG	*Pt×tCSE2*	Real-time PCR
HCT1.RT.for	TTTTATAGGCCCACAGGTGCCT	*Pt×tHCT1*	Real-time PCR
HCT1.RT.rev	CCTGCCATAGGGTAGAATGGT	*Pt×tHCT1*	Real-time PCR
HCT6.RT.for	GGCTGAGACCACCTCAGTTATAGA	*Pt×tHCT6*	Real-time PCR
HCT6.RT.rev	CGCCAGAGTAATCCACAGTAGGT	*Pt×tHCT6*	Real-time PCR
C3'H3.RT.for	CTCAGGCCTTCCTTACTTACAATG	*Pt×tC3'H3*	Real-time PCR
C3'H3.RT.rev	GTAGCCACCAACTTTCACATTGGC	*Pt×tC3'H3*	Real-time PCR
CCoAOMT1.RT.for	AGCCAGGCAGGAAGGCACCAG	*Pt×tCCoAOMT1*	Real-time PCR
CCoAOMT1.RT.rev	CATGCATTCAGGCTCTCTTGGA	*Pt×tCCoAOMT1*	Real-time PCR
CCoAOMT2.RT.for	ACTCAGGCCGGAAGGCATCAA	*Pt×tCCoAOMT2*	Real-time PCR
CCoAOMT2.RT.rev	ATGCATTCAGGCTCTCTTGGG	*Pt×tCCoAOMT2*	Real-time PCR
CCoAOMT3.RT.for	AACTAGTGCATATCCTGGGGAGC	*Pt×tCCoAOMT3*	Real-time PCR
CCoAOMT3.RT.rev	CCTTCATCAACTGGCACAGACATC	*Pt×tCCoAOMT3*	Real-time PCR
CCR7.RT.for	AAACCAGAAGCTAAGGGATCTGGG	*Pt×tCCR2*	Real-time PCR
CCR7.RT.rev	TTCACAGACTCTTCTGCAGCTTG	*Pt×tCCR2*	Real-time PCR
F5H1.RT.for	ACTACTCCGAAGAGGCTGAAACC	*Pt×tF5H1*	Real-time PCR
F5H1.RT.rev	CTCGTTTACTTTTGTCTCTTCACTG	*Pt×tF5H1*	Real-time PCR
F5H2.RT.for	AAGCCAATATAGGCAAGCCTGTGAATC	*Pt×tF5H2*	Real-time PCR
F5H2.RT.rev	ATTTTTAGCCCCGAAAGCTGCTCTG	*Pt×tF5H2*	Real-time PCR
COMT1.RT.for	GGGAATGTCTGACCACTCTACCATTA	*Pt×tCOMT1*	Real-time PCR
COMT1.RT.rev	AGCTCCAGTCCCGCCACCAACAT	*Pt×tCOMT1*	Real-time PCR
COMT2.RT.for	AGCACAATCGTCTCCAAGTACCCT	*Pt×tCOMT2*	Real-time PCR
COMT2.RT.rev	AACATTCTCCACACCAGGGAAAGC	*Pt×tCOMT2*	Real-time PCR
CAD1.RT.for	TGTGGTGAGAATTCCTGATGGG	*Pt×tCAD1*	Real-time PCR
CAD1.RT.rev	CTCTTAGCCCACTCTGTTTCAG	*Pt×tCAD1*	Real-time PCR
CESA1-B.RT.for	GGATGGAGATGAGGATGAAGATGAT	*Pt×tCESA1-B*	Real-time PCR
CESA1-B.RT.rev	ACCCTGCCACTGACGCCTTGCC	*Pt×tCESA1-B*	Real-time PCR
CESA3-A.RT.for	GGTGGATCCCGGAAGAAGAGTTCAG	*Pt×tCESA3-A*	Real-time PCR
CESA3-A.RT.rev	AAGTAGGGTCAACATGCTTGCTCG	*Pt×tCESA3-A*	Real-time PCR
CESA6-A.RT.for	GCACTTATTGTACCACCACACATG	*Pt×tCESA6-A*	Real-time PCR
CESA6-A.RT.rev	TTGGAACCATTGGTCTTGGTTGAG	*Pt×tCESA6-A*	Real-time PCR
CESA6-B.RT.for	GCACTTATTGTTCCACCGAGTC	*Pt×tCESA6-B*	Real-time PCR

CESA6-B.RT.rev	TTGGAACCATTGGTCTTGGTTGAG	*Pt×tCESA6-B*	Real-time PCR
CESA4.RT.for	GGTGCATCCATGCTCCTTTT	*Pt×tCESA4*	Real-time PCR
CESA4.RT.rev	GAACCCACCTTCTAGCAAACTCA	*Pt×tCESA4*	Real-time PCR
CESA7-B.RT.for	GATACATGTGCATCCTGCTTCTAA	*Pt×tCESA7-B*	Real-time PCR
CESA7-B.RT.rev	CATCTCCATCTTAGTCAGTTTATAC	*Pt×tCESA7-B*	Real-time PCR
CESA8-B.RT.for	TTGCTGAGCTACCTCCAATAAG	*Pt×tCESA8-B*	Real-time PCR
CESA8-B.RT.rev	AGGGAAACTACAACGAGGATCA	*Pt×tCESA8-B*	Real-time PCR

References

1. Shi, R. *et al.* Tissue and cell-type co-expression networks of transcription factors and wood component genes in *Populus trichocarpa*. *Planta* **245**, 927–938 (2017).
2. Sundell, D. *et al.* AspWood: High-spatial-resolution transcriptome profiles reveal uncharacterized modularity of wood formation in *Populus tremula*. *Plant Cell* **29**, 1585–1604 (2017).
3. Kumar, V. *et al.* Poplar carbohydrate-active enzymes: whole-genome annotation and functional analyses based on RNA expression data. *Plant J.* **99**, 589–609 (2019).
